# Efficacy of Fu’s Subcutaneous Needling in Treating Soft Tissue Pain of Knee Osteoarthritis: A Randomized Clinical Trial

**DOI:** 10.3390/jcm11237184

**Published:** 2022-12-02

**Authors:** Po-En Chiu, Zhonghua Fu, Jian Sun, Guan-Wei Jian, Te-Mao Li, Li-Wei Chou

**Affiliations:** 1 Department of Chinese Medicine, Chang Bing Show Chwan Memorial Hospital, Changhua 505029, Taiwan; 2 Graduate Institute of Integrated Medicine, College of Chinese Medicine, China Medical University, Taichung 404333, Taiwan; 3 Institute of Fu’s Subcutaneous Needling, Beijing University of Chinese Medicine, Beijing 100029, China; 4 Clinical Medical College of Acupuncture & Moxibustion and Rehabilitation, Guangzhou University of Chinese Medicine, Guangzhou 510405, China; 5 Second Clinical Medical College, Guangzhou University of Chinese Medicine, Guangzhou 510006, China; 6 Guangdong Provincial Hospital of Chinese Medicine, The Second Affiliated Hospital of Guangzhou University of Chinese Medicine, Guangzhou 510260, China; 7 Graduate Institute of Acupuncture Science, China Medical University, Taichung 404333, Taiwan; 8 Department of Chinese Medicine, Sinying Hospital, Ministry of Health and Welfare, Tainan 730020, Taiwan; 9 School of Chinese Medicine, College of Chinese Medicine, China Medical University, Taichung 404333, Taiwan; 10 Department of Physical Medicine and Rehabilitation, China Medical University Hospital, Taichung 404332, Taiwan; 11 Department of Physical Therapy and Graduate Institute of Rehabilitation Science, China Medical University, Taichung 406040, Taiwan; 12 Department of Physical Medicine and Rehabilitation, Asia University Hospital, Asia University, Taichung 413505, Taiwan

**Keywords:** physiotherapy, muscle pain, dry needling, Fu’s subcutaneous needling, acupuncture, knee osteoarthritis, myofascial pain, trigger point, tightened muscle

## Abstract

Purpose: Fu’s subcutaneous needling (FSN) is a new acupuncture technique that produces a long-lasting effect in soft-tissue injuries. In patients with degenerative knee osteoarthritis (OA), myofascial trigger points (MTrPs) are common in the lower-limb muscles. In this randomized clinical trial, we evaluated the immediate, 1-week and 2-week effectiveness of FSN therapy in the treatment of degenerative knee OA. Patients and methods: We randomly divided 32 patients with knee OA into the FSN group (mean age: 65.73 ± 6.79 years) or the transcutaneous electrical nerve stimulation (TENS) group (mean age: 62.81 ± 5.72 years). The pressure pain threshold (PPT) and tissue hardness (TH) of the muscle and tendon attachment sites, knee range of motion, and physical ability (average walking speed) were measured. The subjective pain intensity index, Western Ontario and McMaster Universities OA Index (WOMAC), and Lequesne index were used to determine the efficacy of FSN on MTrP-induced soft-tissue pain compared with that of TENS. Results: A significantly greater improvement in pain qualities in the VAS (*p* < 0.05) was found in the FSN group. Moreover, in muscle and tendon qualities (including PPT and TH), a significant difference in the PPT of the quadriceps muscle (*p* < 0.05) was also observed among the immediate treatments in the FSN group. As for the functional index questionnaire assessment, the FSN group exhibited significant improvements among the immediate, 1-week and 2-week efficacies in terms of WOMAC (*p* < 0.05) and Lequesne index scores (*p* < 0.05). Conclusion: FSN was effective in treating soft-tissue pain in degenerative knee OA in terms of alleviating pain, strengthening walking ability, and improving overall functional performance. Pain relief was the primary benefit of FSN and a significant correlation between pain relief and knee joint mobility improvement was found. Trial registration: ClinicalTrials.gov Protocol Registration and Results System (registration number: NCT04356651).

## 1. Introduction

Osteoarthritis (OA) is the progressive destruction of joint cartilage and a common age-related degenerative joint disease in adults worldwide [1]. One common clinical feature is a loss of cartilage, resulting in pain and partial impairment of joint function, which mainly affects weight-bearing joints such as the wrists, ankles, knees, hips, and vertebrae [2,3]. The incidence of OA has increased considerably in Western industrialized countries, especially in the knee joint [4]. In the United States, the prevalence of degenerative knee OA has increased by approximately twofold since the mid-20th century [5], with more than 13.7 million people estimated to have symptoms of degenerative knee OA, accounting for 6.9% of the total US population over 25 years of age [6]. In Taiwan, according to the Ministry of Health and Welfare, the prevalence of degenerative knee arthritis is approximately 15%, which means that approximately 3.5 million people have the disease and that an average of one in five adults over 58 years of age have degenerative joint problems; more than 70% of adults over 70 years of age have degenerative knee arthritis [7].

No clear pathophysiological explanation for the pathogenesis of degenerative knee OA has been proposed and the symptoms usually progress slowly and often go unnoticed [8]. However, numerous studies have reported considerable discrepancies between patients’ pain levels and their diagnostic imaging findings [9,10]. Reviewed observational studies have suggested a relationship between the pain in muscles related to knee OA and myofascial trigger points (MTrPs) found in the muscles associated with this joint. These may play a key role in the pain of OA [11]. For cases of myofascial pain syndrome in which the source of pain is muscular [12], treating the MTrPs may be an effective method of relieving the pain and dysfunction of degenerative knee arthritis. Needling therapies, such as dry needling in Western medicine and acupuncture in traditional Chinese medicine, are common clinical treatments for MTrP-related disorders [13,14]. Although the efficacy and mechanisms of dry needling for the relief of myofascial pain disorders are not exactly known, numerous studies have demonstrated substantial efficacy. A systematic review and meta-analysis revealed that dry needling reduced pain levels and the incidence of disability in patients with neck pain caused by MTrPs in the short term [15]. Traditional Chinese acupuncture is used to treat myofascial pain by producing a sense of *de qi*, which is a local muscle twitching response to acupuncture [16]. According to clinical studies, acupuncture treatment improves motor function in the knee, relieves pain, improves physical and psychological function in patients with degenerative knee OA, and reduces the risk of requiring artificial knee replacement [17].

Fu’s subcutaneous needling (FSN) is a new technique based on dry needling and traditional acupuncture that provides stimulation primarily in the subcutaneous area and quickly produces a long-lasting effect in soft-tissue injury and other medical conditions [18,19,20]. The treatment involves the use of a disposable FSN needle in the subcutaneous tissues around or adjacent to the affected muscle (so-called pathological “tightened muscle”, with one or more MTrPs in the muscle) [20]. This is usually combined with the swaying movement technique and reperfusion approach for improving symptoms such as abnormal muscle tension, stiffness, pain, limited range of motion, and fatigue.

Clinically, MTrPs in the lower-limb muscles are common in patients with knee OA, but few studies have investigated the effectiveness of the treatment for lower-limb MTrPs in knee OA patients [21]. This study conducted a trial to observe the immediate, 1-week and 2-week effects of FSN therapy in the treatment of degenerative knee OA by using a scientific evaluation process. This study used a pressure algometer to measure the pressure pain threshold (PPT) and tissue hardness (TH) of the muscle and tendon attachment sites, a joint goniometer to measure the angle of knee movement, and an inertial measurement tool with an inertial sensor system to record the average walking speed of the patient’s trajectory after projection. The subjective pain intensity index, the Western Ontario and McMaster Universities OA Index (WOMAC), and a knee arthritis patient pain index (Lequesne index) were used to compare the subjective and objective assessment tools and determine the efficacy of FSN treatment on soft-tissue pain surrounding degenerative knee OA caused by MTrPs.

## 2. Materials and Methods

### 2.1. Study Design

This trial was a randomized, single-blind clinical study, and the trial protocol was approved by the Human Trials Committee and registered with the ClinicalTrials.gov Protocol Registration and Results System (NCT04356651). The protocol for this study was published in detail beforehand [22].

### 2.2. Participants

This study began on 7 July 2020 and ended on 31 December 2020. Patients with degenerative knee OA were recruited from the outpatient clinics of the Department of Rehabilitation and Department of Acupuncture and Moxibustion at the China Medical University Hospital and were screened for eligibility.

The inclusion criteria were as follows: (1) over 50 years of age; (2) unilateral or bilateral knee OA diagnosed based on X-ray films (Kellgren and Lawrence Scale score of above stage 2); (3) with the clinical symptoms of knee pain, only the side with more knee pain was recruited, and MTrPs could be palpated in the quadriceps and gastrocnemius muscles on the side with the most knee pain; and (4) a visual analog scale (VAS) score of >5 for the most painful knee. The exclusion criteria were as follows: (1) general contraindications such as severe internal medical problems, severe recent injury or trauma, or pregnancy; (2) history of drug abuse (including excessive alcohol consumption) that would influence the pain evaluation or current use of anticoagulants (e.g., phenprocoumon and heparin) and coagulopathy in the 12 months prior; (3) infection, ulcers, or injury on the surface of the skin; (4) history of knee operation or replacement; (5) central nervous system diseases or peripheral neuropathy; (6) cognitive dysfunction and inability to participate in the entire trial; and (7) history of other treatments for knee OA, including disease-modifying osteoarthritis drugs, in the 3 months prior.

This was a randomized, single-blind clinical trial, and participants were randomly allocated to either the study group or control group. The study group participants received FSN treatment, whereas the control group participants received transcutaneous electrical nerve stimulation (TENS) treatment. The whole course lasted for 2 weeks. A total of 3 treatment sessions in this experiment were performed in the first week with assessment before each treatment session and immediately after treatment as well as in the following 1st and 2nd weeks of follow-up. All the treatments were conducted by the same acupuncturist who worked in the medical center for more than five years. The participants provided informed consent before the trial. Each participant’s basic information, such as gender, age, occupation, address, telephone number, and medical history, was noted prior to the trial to ensure their privacy. Sociodemographic data, such as age, gender, side of pain, height, and weight, were collected for each participant prior to treatment.

### 2.3. Outcome Measurements

A single-blind experiment was conducted. Because the participants and the individual administering the treatment could not be blinded, the treatment was not administered by the assessor so the assessor would be unaware of the grouping of the participants. The assessor was also instructed to not communicate with the participants about their treatment.

The outcome measurements were collected by the assessor, including the pain qualities, the muscle and tendon qualities, and the functional index questionnaire assessment. For the pain qualities, the VAS, PPT of the quadriceps muscle, walking speed, and ROM of the knee were assessed. The participants described the intensity of pain around their knees before and after each treatment, which was expressed using the VAS as the subjective pain intensity. A semi-objective assessment tool, the Tissue Hardness Meter/Algometer Combo (OE-220, ITO Co., Ltd., Tokyo, Japan), was used to measure the PPT at the quadriceps. During the experiment, the method recommended by Fischer [23,24,25] was used to measure the PPT before and after each treatment session, and 1 and 2 weeks thereafter. The inertial measurement tool used for measuring the walking speed was a Microgate product (GYKO accelerometer, Microgate, Bolzano, Italy) with a high-speed three-dimensional accelerometer and gyroscope [26,27]. Before and after each treatment, the participants wore the device on the upper body below the scapulae by using a special bib to acquire information regarding the participants’ trunk movement on the ground. The average walking speed was measured before and after each treatment session, and 1 and 2 weeks thereafter. Active range of motion (AROM) and passive range of motion (PROM) [28,29,30] were measured using a joint goniometer before and after each treatment, and 1 and 2 weeks thereafter.

The PPT and TH were assessed as the muscle and tendon qualities. A semi-objective assessment tool was used to measure the PPT at three locations, namely, the quadriceps muscle, pes anserinus tendon, and gastrocnemius muscle. During the experiment, the PPT was measured before and after each treatment session, and 1 and 2 weeks thereafter. TH [31,32,33] was measured at the same locations and timescales as the PPT using a semi-objective assessment tool with the same machine that was used for the Tissue Hardness Meter/Algometer Combo (OE-220, ITO Co., Ltd.).

The WOMAC and Lequesne indices were assessed as the functional index questionnaires. Knee structure and function were assessed using the WOMAC in terms of pain, stiffness, and joint function in the lower limbs [34]. The participants completed the test before the first treatment, and 1 and 2 weeks after the treatment; the scores for each subscale were summed up with a possible score range of 0–20 for Pain, 0–8 for Stiffness, and 0–68 for Physical Function. Usually, a sum of the scores for all three subscales gives a total WOMAC score; higher scores on the WOMAC indicate worse pain, stiffness, and functional limitations. The Lequesne index consists of three parts: pain and discomfort; walking distance; and daily joint mobility [35]. The scoring is from 0 (no pain or disability) to 24 (maximal pain and disability). The participants completed these two questionnaires before the first treatment and 1 and 2 weeks after the treatment.

### 2.4. Procedure and Intervention

#### 2.4.1. Selection of Needle Site for Experimental Group (FSN Group)

**Figure 1:**
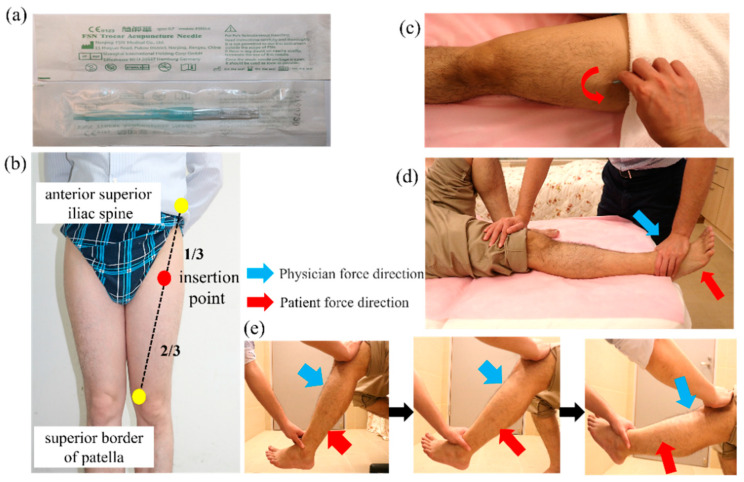
Illustration of the operations of Fu’s subcutaneous needling. FSN was performed as described in the Materials and Methods. (**a**) FSN needle. (**b**) Insertion point of FSN insertion. (**c**) Starting a swaying movement. (**d**) Dorsiflexion of the foot and resistance towards the practitioner in the reperfusion approach. (**e**) Knee extension and physician’s resistance in the reperfusion approach.

Disposable FSN needles (Figure 1a) produced by Nanjing Paifu Medical Science and Technology Co. (Nanjing, China) were used. The participants lay on their backs with the examined knee straight and the pelvis in a neutral position. The needle was inserted at the 1/3 spot from the connecting line between the anterior superior iliac spine to the proximal of the superior border of the patella (Figure 1b). The needle was completely inserted toward the patella until it was fully submerged into the subcutaneous tissue. To ensure that the needle did not enter the dermis or muscle layer during needling, the participants were asked to indicate if they felt pain. Then, the needle core was retracted into the hose and swung from side to side, the so-called “swaying movement” (Figure 1c). The needle was advanced approximately 60° to the left and right (so-called swaying movement), and a total of 45 round trips were carried out in 30 s. Participants were also asked to indicate pain during this process. The patients were asked to perform a 1-min cycle of dorsiflexion of the feet, with 10 s of rest after 10 s of movement. Then, the participants were asked to sit and perform a similar 1-min cycle of flexing and extending their knees and resting for 10 s after 10 s of continuous movement. The above actions are called the “reperfusion approach” (Figure 1d,e). Finally, the needle was removed and a dry cotton ball with adhesive tape was applied to the hole made by the needle to prevent bleeding.

#### 2.4.2. Selection of Electrical Stimulation Points for Control Group (TENS Group)

**Figure 2:**
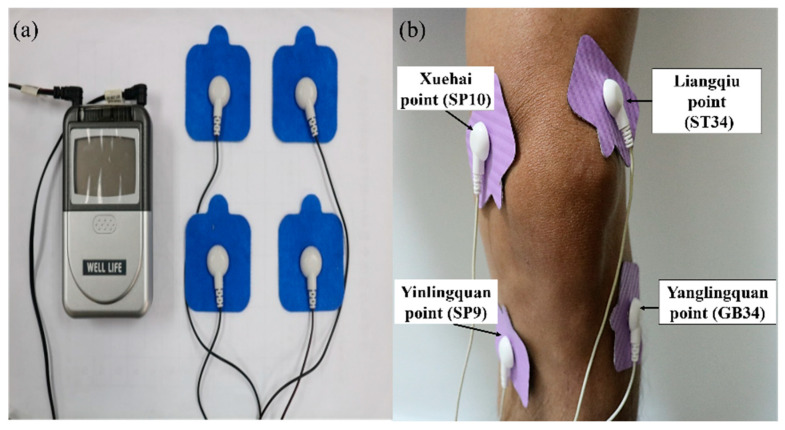
Illustration of the transcutaneous electrical nerve stimulation (TENS). (**a**) TENS. (**b**) TENS pads were attached at ST34, GB34, SP10, and SP9.

The device for TENS [36] is manufactured by Well-Life Healthcare (Model Number 2205A, New Taipei City, Taiwan) (Figure 2a). A patch on the skin is applied for electrical nerve stimulation of various frequencies and intensities of the affected area to relieve pain. Electrode placements are applied to the Liangqiu (ST34) and Yanglingquan (GB34) points on the lateral side of the knee cap and the Xuehai (SP10) and Yinlingquan (SP9) points on the medial side. The current is then passed through each patch and across the knee joint (Figure 2b). On the basis of other studies [37,38,39], we selected a treatment consisting of a continuous wave (ADJ waveform) with a pulse frequency of 110 Hz. The treatment lasts 20 min, after which the machine is turned off and the electrode patches are removed.

### 2.5. Statistical Analysis

Statistical analysis was performed using SPSS (version 22.0, IBM SPSS Statistics, IBM Corp. Released 2013. IBM SPSS Statistics for Windows, Version 22.0. Armonk, NY, USA: IBM Corp). The continuous variables were the VAS, PPT, average walking speed, range of motion, TH, WOMAC, and Lequesne index scores. All the data are presented as the means ± standard deviations and were analyzed through a *t*-test. Statistical significance was set at *p* < 0.05.

The immediate effects were the changes in the variables immediately after each treatment. The 1-week effect was identified by comparing the variables before the first treatment with those 1 week after treatment. The 2-week effect was identified by comparing the variables before the first treatment with those 2 weeks after treatment. A paired *t*-test was used for intragroup comparison, and an independent *t*-test was used for intergroup comparison.

## 3. Results

**Figure 3:**
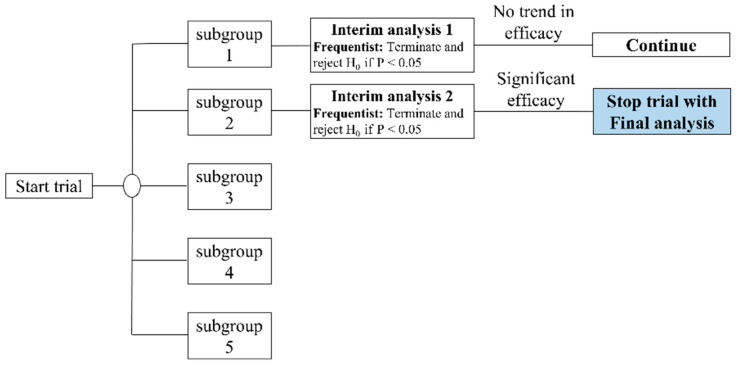
Flow chart of the adaptive study design with an interim analysis.

**Figure 4:**
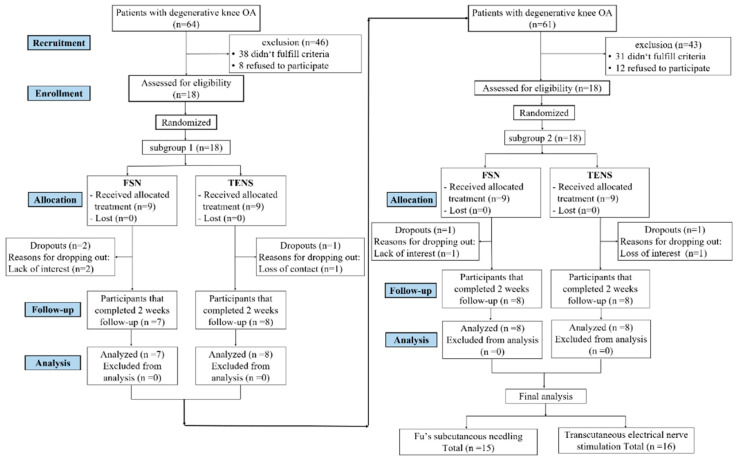
Flow chart summarizing follow-up on clinical outcomes and treatment preferences.

From July 2020 to December 2020, we screened 125 patients with knee OA and 36 of them who met the inclusion criteria were included and randomly assigned. The original estimate was 90 subjects in our study protocol [22]. We called for early trial termination in order to conduct the interim analysis [40]. We divided our participants into five subgroups, with 18 subjects in each subgroup, and then divided all the participants into random groups. Each intake of 18 participants was preceded by an interim analysis (Figure 3). There was no statistically significant difference in the first subgroup. Three participants were lost at the end of the first follow-up period (two in the FSN group and one in the TENS group). Regarding the second subgroup, although two participants were lost (one in the FSN group and one in the TENS group), a statistically significant difference was found. As a result, we terminated the participant recruitment project early. In total, 31 participants (10 male and 21 female) completed the study, with 15 in the FSN group (4 male and 11 female) and 16 in the TENS group (6 male, 10 female) (Figure 4).

### 3.1. Study and Patient Characteristics

Table 1 presents the participants’ baseline characteristics. The mean age in the FSN group was 65.73 ± 6.79 years old and that in the TENS group was 62.81 ± 5.72 years old. No significant differences in baseline demographic or clinical characteristics between these two groups were observed (*p* > 0.05).

### 3.2. Pain Qualities

Table 2 shows the VAS, PPT of the quadriceps muscle, walking speed, and ROM of the knee before and after each pre-treatment (pre-tx), and 1 and 2 weeks post-treatment (post-tx), in the FSN and TENS groups, where a *p*-value of less than *0.05* is statistically *significant*.

**Figure 5:**
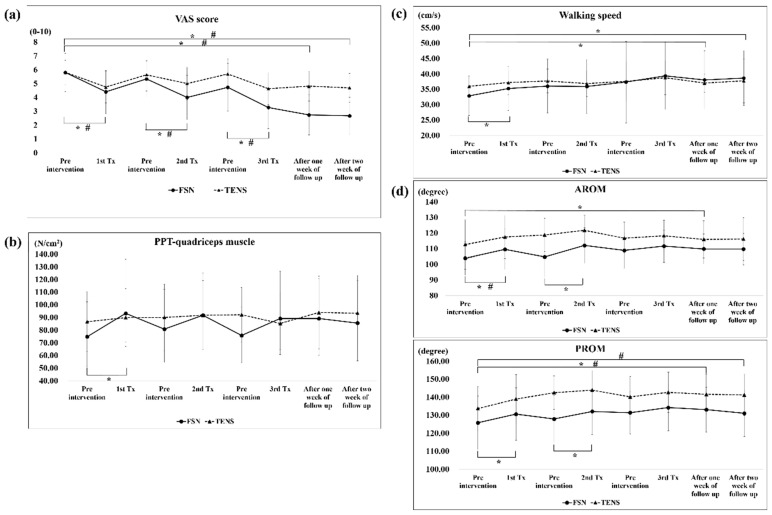
Comparison of the VAS, PPT of the quadriceps muscle, walking speed, and ROM between the two groups after each treatment. The pre- and post-treatment values of the VAS (**a**), PPT-quadriceps muscle (**b**), walking speed (**c**), and ROM (**d**) were measured in 3 treatment sessions in both groups and followed up after one week and two weeks in both groups. * represents the FSN group *p* < 0.05; # represents the TENS group *p* < 0.05. VAS: visual analog scale; PPT: pressure pain threshold; AROM: active range of motion; PROM: passive range of motion; FSN: Fu’s subcutaneous needling; TENS: transcutaneous electrical nerve stimulation.

The analysis of the overall efficacy of the FSN and TENS treatments over time revealed a significant difference in VAS scores among the immediate (FSN: Day 1 pre-tx, 5.80 ± 1.42, post-tx, 4.40 ± 1.58; TENS: Day 1 pre-tx, 5.81 ± 0.91, post-tx, 4.75 ± 1.15; FSN: Day 2 pre-tx, 5.33 ± 1.30; post-tx, 4.00 ± 1.59; TENS: Day 2 pre-tx, 5.63 ± 1.17, post-tx, 5.00 ± 1.17; FSN: Day 4 pre-tx, 4.73 ± 1.73; post-tx, 3.27 ± 1.53; TENS: Day 4 pre-tx, 5.69 ± 1.10, post-tx, 4.63 ± 1.17), 1-week (FSN: post-tx, 2.73 ± 1.44; TENS: post-tx, 4.81 ± 1.07), and 2-week effects (FSN: post-tx, 2.67 ± 1.35; TENS: post-tx, 4.69 ± 1.04) at any given time (Figure 5a).

As for the PPT of the quadriceps muscle, no significant difference was observed among the groups, except for the first treatment of the FSN group (FSN: Day 1 pre-tx, 74.73 ± 28.63; post-tx, 93.20 ± 42.84) (Figure 5b).

As for the walking speed, a significant difference was observed between the first treatment (pre-tx, 32.80 ± 6.60 cm/s; post-tx, 35.25 ± 7.18 cm/s) and 1-week (post-tx, 38.02 ± 9.41 cm/s) and 2-week outcomes (post-tx, 38.61 ± 8.94 cm/s) in the FSN group, but not in the TENS group (Figure 5c).

A significant difference in AROM and PROM was observed among the first treatment (FSN, AROM: pre-tx, 103.87 ± 11.17; post-tx, 109.53 ± 12.86; PROM: pre-tx, 125.80 ± 15.33; post-tx, 130.60 ± 14.55), second treatment (FSN, AROM: pre-tx, 104.67 ± 12.85; post-tx, 112.07 ± 11.34; PROM: pre-tx, 127.87 ± 13.05; post-tx, 132.07 ± 12.94), and 1-week outcome (FSN, AROM: post-tx, 109.80 ± 9.55; PROM: PROM: post-tx, 133.07 ± 12.42). Compared with the TENS group, a significant difference was observed among the first treatment (TENS, AROM: pre-tx, 112.63 ± 16.31; post-tx, 117.44 ± 13.73), 1-week outcome (TENS, PROM: post-tx, 141.56 ± 11.32), and 2-week outcome (TENS, PROM: post-tx, 141.25 ± 11.50) (Figure 5d).

### 3.3. Muscle and Tendon Qualities

Table 3 shows the PPT and TH of the quadriceps muscle, pes anserinus, and gastrocnemius muscle before and after each treatment, and 1 and 2 weeks post-treatment, in the FSN and TENS groups, where a *p*-value of less than 0.05 is statistically significant.

**Table 2:** Pain qualities (including VAS, PPT of quadriceps muscle, walking speed, and ROM of knee) compared between the FSN and TENS groups.

	Day 1			Day 2			Day 4			Day 8		Day 15	
	Pre-tx	Post-tx	*P*	Pre-tx	Post-tx	*P*	Pre-tx	Post-tx	*P*	1-Week F/U	*P*	2-Week F/U	*P*
VAS													
FSN group	5.80 ± 1.42	4.40 ± 1.58	<0.05 *	5.33 ± 1.30	4.00 ± 1.59	<0.05 *	4.73 ± 1.73	3.27 ± 1.53	<0.05 *	2.73 ± 1.44	<0.05 *	2.67 ± 1.35	<0.05 *
TENS group	5.81 ± 0.91	4.75 ± 1.15	<0.05 *	5.63 ± 1.17	5.00 ± 1.17	<0.05 *	5.69 ± 1.10	4.63 ± 1.17	<0.05 *	4.81 ± 1.07	<0.05 *	4.69 ± 1.04	<0.05 *
PPT of quadriceps muscle (N/cm^2^)													
FSN group	74.73 ± 28.63	93.20 ± 42.84	<0.05 *	80.80 ± 31.40	91.80 ± 33.42	0.06	75.73 ± 22.51	89.13 ± 37.55	0.13	89.13 ± 31.37	0.07	85.60 ± 33.37	0.31
TENS group	86.56 ± 24.35	89.88 ± 22.76	0.57	90.06 ± 26.26	91.81 ± 27.35	0.66	92.13 ± 21.66	85.31 ± 24.61	0.08	93.88 ± 28.77	0.18	93.44 ± 29.95	0.24
Walking speed (cm/s)													
FSN group	32.80 ± 6.60	35.25 ± 7.18	<0.05 *	36.00 ± 8.76	35.87 ± 8.87	0.96	37.34 ± 13.25	39.34 ± 10.94	0.36	38.02 ± 9.41	<0.05 *	38.61 ± 8.94	<0.05 *
TENS group	35.96 ± 3.20	37.16 ± 3.90	0.15	37.66 ± 3.88	36.84 ± 4.23	0.50	37.57 ± 6.51	38.74 ± 5.57	0.44	36.98 ± 4.98	0.47	37.74 ± 7.17	0.38
AROM of knee (degrees)													
FSN group	103.87 ± 11.17	109.53 ± 12.86	<0.05 *	104.67 ± 12.85	112.07 ± 11.34	<0.05 *	108.87 ± 11.45	111.60 ± 10.30	0.27	109.80 ± 9.55	<0.05 *	109.73 ± 10.02	0.09
TENS group	112.63 ± 16.31	117.44 ± 13.73	<0.05 *	118.81 ± 10.64	121.75 ± 9.59	0.29	116.63 ± 10.33	118.25 ± 9.91	0.76	115.94 ± 11.85	0.57	116.13 ± 13.66	0.55
PROM of knee (degrees)													
FSN group	125.80 ± 15.33	130.60 ± 14.55	<0.05 *	127.87 ± 13.05	132.07 ± 12.94	<0.05 *	131.40 ± 11.80	134.20 ± 12.90	0.23	133.07 ± 12.42	<0.05 *	131.07 ± 12.90	0.25
TENS group	135.69 ± 16.80	138.94 ± 13.60	0.12	142.50 ± 9.26	143.94 ± 10.83	0.27	140.19 ± 11.32	142.69 ± 11.22	0.06	141.56 ± 11.32	<0.05 *	141.25 ± 11.50	<0.05 *

Data are expressed as the mean ± SD; * indicates a significant difference, analyzed by a paired *t*-test. Abbreviations: FNS, Fu’s subcutaneous needling; TENS, transcutaneous electrical nerve stimulation; VAS, visual analog scale; PPT, pain pressure threshold; AROM, active range of motion; PROM, passive range of motion.

**Table 3:** Muscle and tendon qualities (including PPT and TH) compared between the FSN and TENS groups.

	Day 1			Day 2			Day 4			Day 8		Day 15	
	Pre-tx	Post-tx	*P*	Pre-tx	Post-tx	*P*	Pre-tx	Post-tx	*P*	1-Week F/U	*P*	2-Week F/U	*P*
PPT of quadriceps muscle (N/cm^2^)													
FSN group	74.73 ± 28.63	93.20 ± 42.84	<0.05 *	80.80 ± 31.40	91.80 ± 33.42	0.06	75.73 ± 22.51	89.13 ± 37.55	0.13	89.13 ± 31.37	0.07	85.60 ± 33.37	0.31
TENS group	86.56 ± 24.35	89.88 ± 22.76	0.57	90.06 ± 26.26	91.81 ± 27.35	0.66	92.13 ± 21.66	85.31 ± 24.61	0.08	93.88 ± 28.77	0.18	93.44 ± 29.95	0.24
PPT of pes anserinus (N/cm^2^)													
FSN group	66.53 ± 34.06	61.47 ± 26.26	0.75	60.87 ± 28.69	65.93 ± 23.37	0.16	63.80 ± 25.19	66.07 ± 25.65	0.33	61.60 ± 22.81	0.73	65.40 ± 22.55	0.22
TENS group	63.38 ± 17.76	64.25 ± 17.79	0.78	72.06 ± 25.33	72.44 ± 24.35	0.87	68.00 ± 21.48	67.25 ± 19.51	0.78	71.00 ± 24.01	0.36	76.44 ± 24.08	<0.05 *
PPT of gastrocnemius muscle (N/cm^2^)													
FSN group	88.47 ± 34.05	88.87 ± 37.90	0.87	84.13 ± 32.79	94.67 ± 34.56	0.16	93.73 ± 29.34	89.07 ± 29.12	0.37	91.53 ± 24.25	0.49	87.93 ± 20.03	0.69
TENS group	82.13 ± 24.97	91.06 ± 26.58	<0.05 *	92.75 ± 27.66	89.19 ± 24.62	0.28	93.50 ± 28.64	88.13 ± 29.51	0.13	92.38 ± 37.91	0.24	87.38 ± 30.41	0.78
TH of quadriceps muscle (N)													
FSN group	47.79 ± 10.04	45.89 ± 7.11	0.26	45.55 ± 6.64	45.98 ± 7.29	0.91	46.01 ± 5.86	42.87 ± 6.04	0.31	42.99 ± 6.29	0.07	46.73 ± 10.94	0.61
TENS group	45.39 ± 9.51	51.31 ± 8.47	<0.05 *	47.36 ± 10.33	49.67 ± 7.73	0.29	51.05 ± 9.92	52.66 ± 8.22	0.76	46.79 ± 7.93	0.57	47.61 ± 6.64	0.55
TH of pes anserinus (N)													
FSN group	41.44 ± 9.94	44.92 ± 10.90	0.39	41.23 ± 9.12	39.75 ± 8.19	0.69	39.61 ± 11.14	41.17 ± 9.64	0.31	40.23 ± 13.78	0.32	42.49 ± 11.95	0.78
TENS group	37.80 ± 4.40	34.81 ± 9.98	0.33	37.16 ± 7.32	38.59 ± 9.15	0.45	36.76 ± 13.40	38.47 ± 11.72	0.65	34.41 ± 12.89	0.24	31.57 ± 13.22	0.09
TH of gastrocnemius muscle (N)													
FSN group	36.79 ± 6.30	39.75 ± 8.70	0.50	39.15 ± 8.46	39.67 ± 7.98	0.80	38.13 ± 10.18	41.15 ± 10.02	0.29	37.13 ± 6.50	0.82	36.04 ± 8.16	0.69
TENS group	35.15 ± 6.97	37.87 ± 9.47	0.26	35.29 ± 7.71	35.71 ± 6.33	0.57	38.08 ± 6.99	34.84 ± 6.72	0.07	39.13 ± 8.04	0.07	38.05 ± 5.77	0.16

Data are expressed as the mean ± SD; * indicates a significant difference, analyzed by a paired *t*-test. Abbreviations: FNS, Fu’s subcutaneous needling; TENS, transcutaneous electrical nerve stimulation; PPT, pain pressure threshold; TH, tissue hardness of muscle.

**Figure 6:**
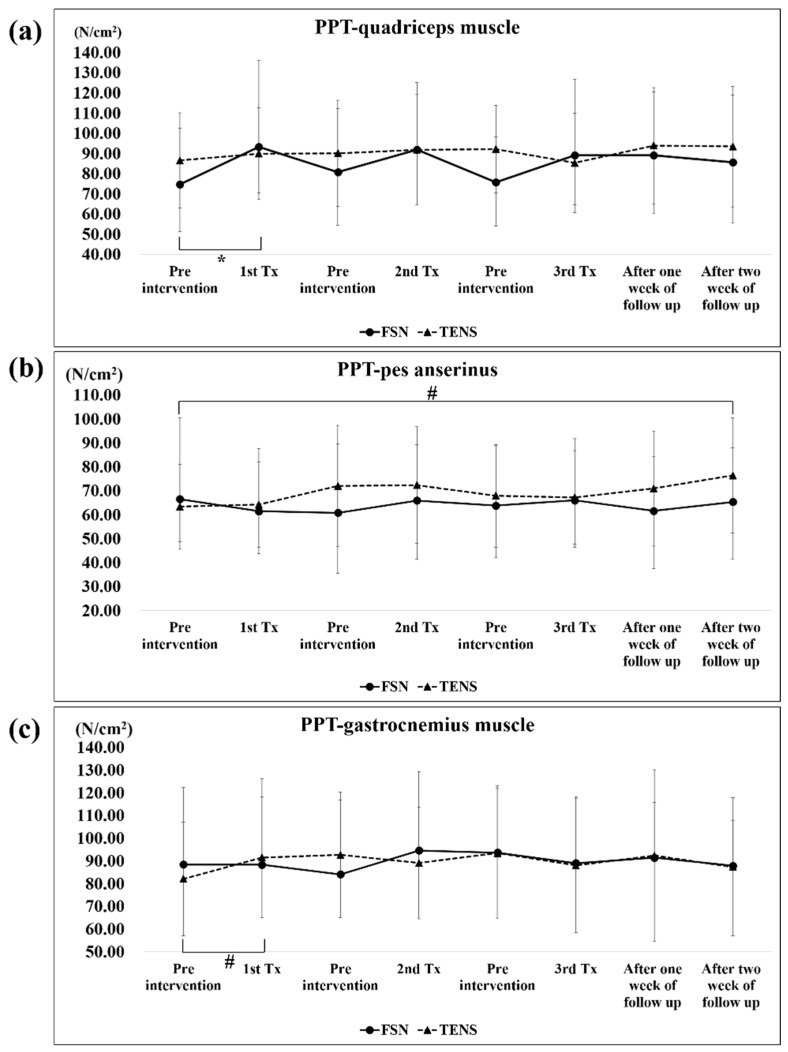
Comparison of the PPT of the quadriceps muscle (**a**), pes anserinus (**b**), and gastrocnemius muscle (**c**) between the two groups after each treatment. * represents the FSN group *p* < 0.05; # represents the TENS group *p* < 0.05. PPT: pressure pain threshold; FSN: Fu’s subcutaneous needling; TENS: transcutaneous electrical nerve stimulation.

A significant difference in the PPT of the quadriceps muscle was only observed in the first FSN treatment (pre-tx, 74.73 ± 28.63; post-tx, 93.20 ± 42.84); no statistically significant differences were noted in the TENS groups. As for the pes anserinus, a significant difference was only observed in the 2-week outcomes (post-tx, 76.44 ± 24.08) in the TENS group but not in the FSN group. As regards the gastrocnemius muscle, a significant difference was only observed in the first TENS treatment (pre-tx, 82.13 ± 24.97; post-tx, 91.06 ± 26.58); no statistically significant differences were noted in the FSN group (Figure 6a–c).

**Figure 7:**
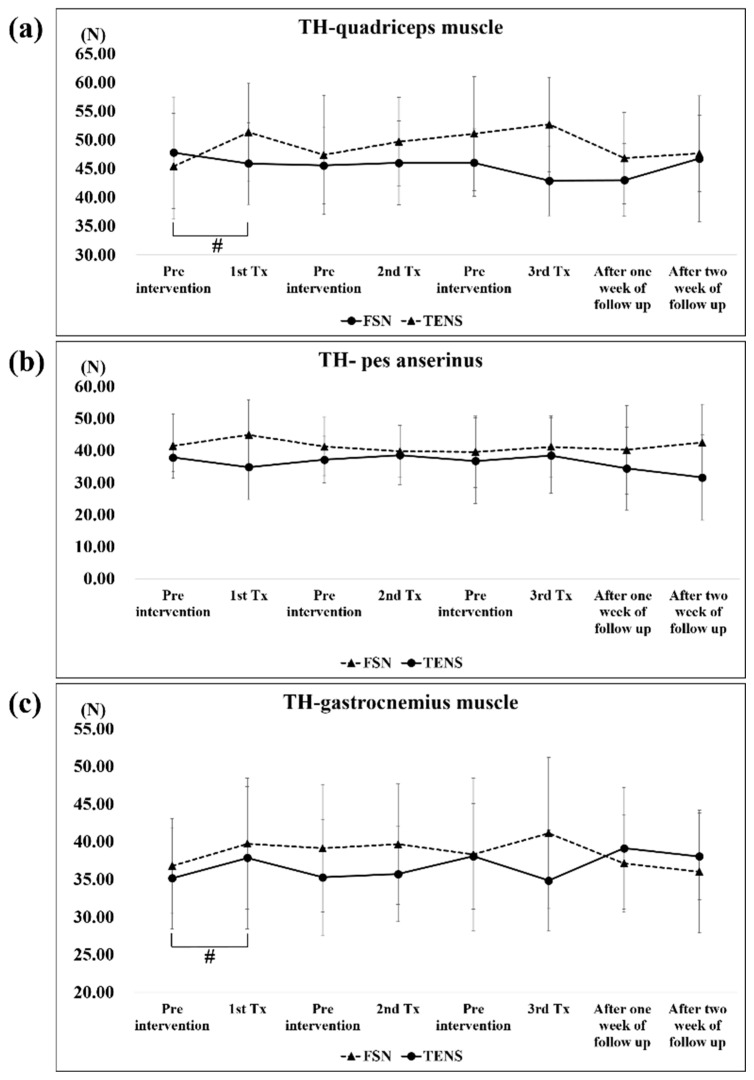
Comparison of TH in the quadriceps muscle (**a**), pes anserinus (**b**), and gastrocnemius muscle (**c**) between the two groups after each treatment. # represents the TENS group *p* < 0.05. TH: tissue hardness of muscle; FSN: Fu’s subcutaneous needling; TENS: transcutaneous electrical nerve stimulation.

A significant difference in the TH of the quadriceps muscle was only observed in the first TENS treatment (pre-tx, 45.39 ± 9.51; post-tx, 51.31 ± 8.47). There were no significant changes in the TH of the pes anserinus and gastrocnemius muscles in both groups. In the FSN group, a slight decrease in tissue hardness was observed in the quadriceps muscle after each treatment, which then increased again before the next treatment, showing a sawtooth-like improvement, while the rest of the group showed no such improvement. In the TENS group, for the quadriceps muscle, there was a significant increase after each TENS treatment due to electrical stimulation (Figure 7a–c).

### 3.4. Functional Index Questionnaire Assessment

Table 4 shows the WOMAC and Lequesne indices before and after the treatment, and 1 and 2 weeks post-treatment in the FSN and TENS groups, where a *p*-value of less than 0.05 is statistically significant.

**Figure 8:**
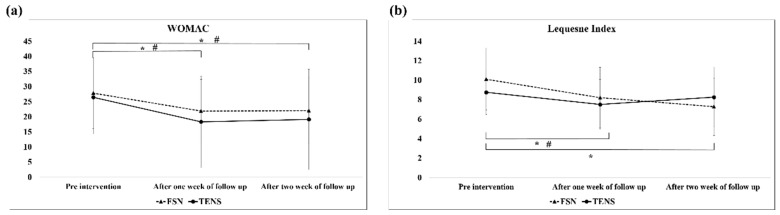
Comparison of WOMAC (**a**) and Lequesne index scores (**b**) between the two groups after each treatment. * represents the FSN group *p* < 0.05; # represents the TENS group *p* < 0.05. WOMAC: Western Ontario and McMaster Universities Osteoarthritis Index; FSN: Fu’s subcutaneous needling; TENS: transcutaneous electrical nerve stimulation.

The analysis of the WOMAC scores of the overall efficacy of the FSN and TENS treatments over time revealed a significant difference between the two groups of the 1-week follow-up (FSN: post-tx, 21.87 ± 10.50; TENS: post-tx, 18.31 ± 15.02) and 2-week follow-up (FSN: post-tx, 22.00 ± 11.06; TENS: post-tx, 19.13 ± 16.64) at any given time. As for the Lequesne index score, a significant difference was observed only between the 1-week (post-tx, 8.20 ± 3.12) and 2-week outcomes (post-tx, 7.27 ± 2.95) in the FSN group; however, in the TENS group, a significant difference was only revealed in the 1-week outcome (post-tx, 7.50 ± 2.57) (Figure 8). Significant differences in the 1- and 2-week WOMAC effects were also observed between the two groups (Figure 8a). In addition, significant differences were observed in efficacy in the Lequesne index group between weeks 1 and 2 and in efficacy in the control group between weeks 1 and 2 (Figure 8b).

## 4. Discussion

To our knowledge, our study is the first randomized clinical trial comparing the effectiveness of FSN and TENS alone in patients with painful knee OA. We discuss this through three perspectives: pain qualities, muscle and tendon qualities, and functional index questionnaire assessment. The results of our study indicated that FSN reduced the pain significantly and the quadriceps muscle showed an immediate improvement in the PPT after each FSN treatment. As for the functional index questionnaire assessment, our research indicated that the treatment effects of FSN persisted for longer than those of TENS. These results indicate that FSN is an effective treatment for knee OA.

In recent years, many studies have shown that muscle dysfunction is the key issue for the development and progression of knee OA and MTrPs are a common cause of musculoskeletal pain conditions [41]. As a result, attention has begun to be focused on the soft tissues surrounding the knee joint. TENS was shown by many studies to be a safe and effective treatment for knee OA and it is also known as an effective pain management modality for OA-related knee pain. Vance et al. [36] noted that the effects of TENS on pain reduction are related to its effects on hyperalgesia through both peripheral and central mechanisms. This mechanism provides immediate pain relief caused by MTrPs [42]. FSN is considered one type of dry needling. In recent years, FSN therapy has been widely used in the treatment of pain-related musculoskeletal disorders [43]. Although the potential mechanism of FSN treatment remains unclear, it is generally accepted that MTrPs are a common cause of musculoskeletal pain; therefore, they have become the theoretical basis for much of the treatment [44]. FSN adopted this theory, where MTrPs were originally identified as a treatment target.

### 4.1. Pain Qualities (Including VAS, PPT, Walking Speed, and ROM of Knee)

Our clinical trial study demonstrated that subjective pain perception was reduced in both the FSN and TENS groups. We also observed that the pain level in the FSN group improved in most cases; however, the pain level gradually increased after the previous treatment. Similarly, the pain level before the second treatment was less than before the first treatment, and the pain level before the third treatment was between that before and after the second treatment, indicating a jagged decrease (Figure 5a), which is similar to most of the general patterns of pain treatment. In terms of reducing subjective pain perception, the FSN treatment was more effective; the gap in the line graph (Figure 5a) becomes more obvious over time. In objective experimental data, the PPT is used to measure the tolerance of intramuscular MTrPs to external pressure stimulation, also known as pain tolerance. A low PPT indicates a low tolerance. The line graph (Figure 5b) indicated that the PPT of the MTrPs in the quadriceps muscle increased after each treatment in the FSN group. Because the quadriceps muscle segment was treated with FSN in the experimental group, the local muscle tissue of the quadriceps muscle may have improved more after the treatment and the pain threshold of the MTrPs measured in the quadriceps muscle belly increased accordingly. The jagged decrease in the VAS corresponded to the increase in the jaggedness of the PPT. When the quadriceps muscle was treated with FSN, the muscle tissue improved to a greater extent, and the PPT at the MTrPs measured in the quadriceps muscle belly improved. As a result, the patient’s subjective VAS was reduced.

The line graph for average walking speed (Figure 5c) indicated that patients’ average walking speed increased after each FSN treatment. The first- and second-week post-treatment in the FSN group showed significant improvements because FSN reduced stiffness in the muscle tissue and relieved pain. The difference between the FSN group and the TENS group in the 1-week and 2-week effects in the line graphs (Figure 5c) progressively increases, demonstrating a greater improvement in the effectiveness of the FSN treatment. We already know that a slower walking speed is associated with incident knee OA and that decreasing walking speed has been suggested as one of the mechanisms to reduce pain in the knee [45]. However, according to previous studies, walking speed can be increased through specific exercise therapy programs or physiotherapy to improve the muscles around the knee joint [46,47]; our result is in agreement with the results of previous studies.

Furthermore, we measured the AROM and PROM of the treated knees separately. ROM increased after each FSN treatment and decreased before the subsequent treatment. Up to the 2-week follow-up, significant improvement was still observed after the FSN treatment. Because pain in the knee joint affects the knee movement angle, knee movement is correlated with patients’ pain level, as well as restricted knee function. Because TENS is only a localized pain relief therapy, the reperfusion approach with FSN treatment had a similar effect to the passive joint movement and muscle energy technique in physiotherapy, that is, increasing the extensibility of the tissues surrounding the knee joint, restoring the relationship between the length and tension of the muscles controlling joint movement, stretching tense muscles and fascia, reducing muscle tonus, improving local blood circulation, strengthening weak muscles, and increasing the angle of joint movement [48,49].

Inactivating MTrPs around the knee joint could reduce tension within the muscles and improve mechanical balance, thus relieving joint pain, and control of knee pain. Rahbar et al. [50] divided knee OA patients into two groups, with the control group undergoing 16 types of conventional physiotherapy and the intervention group targeting areas around the knee where MTrPs were present. The results showed that the intervention group recovered from knee OA pain to a greater extent than the control group. In our study, FSN was used to stimulate the MTrPs by performing a swaying movement under the skin to loosen the connective tissue, which similarly restored the tissue structure and improved the mechanical balance of the knee by changing the fascial tension [51].

### 4.2. Muscle and Tendon Qualities

As discussed previously, the PPT in the quadriceps muscle but not the gastrocnemius muscle and pes anserinus, increased after each treatment in the FSN group. The pes anserinus (“goose foot”) refers to the conjoined tendons of three muscles of the thigh; it is attached to the tibia. Because the target of FSN treatment is mainly the MTrPs in the muscles, no direct treatment exists for tendons. Thus, no direct statistical difference in the PPT of the pes anserinus tendon in each treatment of FSN was observed. The gastrocnemius muscle is at a more distal distance from the treatment area; therefore, it presents a tendency to be less variable, which is consistent with the original experimental design of the FSN treatment targeting the affected muscle MTrP.

We learned that the MTrP in the taut band is caused by a shortening of the sarcomere, which should cause muscle tightness and presumably an increase in TH. In previous studies, TrP injection was found to cause changes in TH, therefore it might be meaningful to assume that TH could be used to observe changes in MTrPs after treatment with FSN or TENS [52]. However, in our study, we found that the TH of the quadriceps muscle decreased after each treatment and increased before subsequent treatments but non-significantly. The other two muscles were stretched to 2 weeks before and after treatment with no significant difference in efficacy, where it was expected that the tightened muscle would become soft after FSN and hard after electrical stimulation. Because the pes anserinus belongs to the sartorius muscle, gracilis muscle, and semitendinosus muscle at the point of the tibial attachment of the knee, and because the experimental group’s FSN was administered to increase the TH of the muscle belly, no increase in the TH of the pes anserinus was observed. Because the gastrocnemius muscle was far from the location of the treatment, the difference in TH was small. Another possible reason for the lack of a significant increase in TH with FSN or TENS is that values measured using a myotonic gauge may include both the subcutaneous fat layer and the TH. According to a study on temporomandibular disorders and masticatory myofascial pain, this makes experiments prone to errors and increases the difficulty in obtaining actual values through measurement; this affected the results of this study [53].

### 4.3. Functional Index Questionnaire Assessment

In our study, the WOMAC scores (Figure 8a) decreased in the first week and increased slightly in the second week because the patients were treated three times in the first week, which immediately relieved the pain and stiffness in their knees. We also found that the efficacy of the FSN treatment persisted for longer than that of the TENS treatment but the variance in the data during the first week was higher in the control group than in the FSN group. Because each item on the WOMAC questionnaire begins with the question, “How difficult is it for you to experience pain, discomfort, or arthritis?”, some studies have suggested that this questionnaire does not clearly distinguish between pain and functional structures [54]. The Lequesne questionnaire results indicated a significant difference in the FSN group in the first and second weeks and a significant difference in the TENS group in the first week but not in the second week, indicating that both FSN and TENS relieved pain in patients with knee OA immediately and that the relief lasted in the FSN group but not in the TENS group. The Lequesne index is a 10-question survey of patients with knee OA: five questions related to pain and discomfort, one question related to maximum walking distance, and four questions related to activities of daily living. This indicates that FSN can both reduce pain and increase distance and joint mobility for a long period. The line graph indicated that, although both groups exhibited improvement, the efficacy in the FSN group increased over time despite the fact that no additional treatment was administered in the second week. This might be because the local muscle tissue was in the process of returning to its normal state.

**Figure 9:**
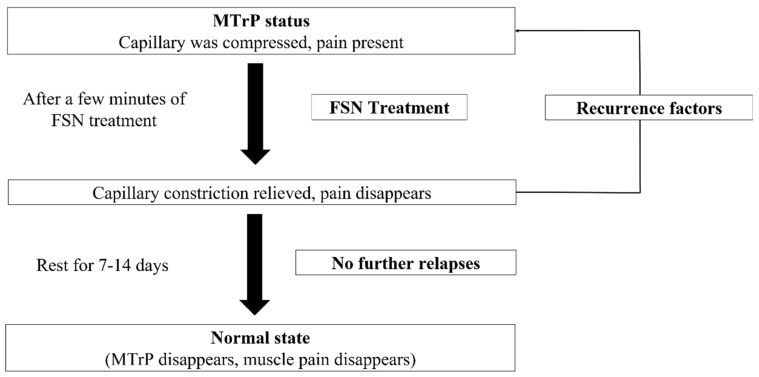
Improvement process for MTrPs.

In our research results, we selected the quadriceps muscle belly for the FSN treatment. Although the local pressure point of the knee (tibial attachment of the pes anserinus tendon) did not improve, the overall knee symptoms and function (VAS, mean walking speed, ROM, WOMAC, Lequesne index) improved. This means that the pain site is not the actual site of the lesion. The actual site is the source of the symptoms in the other parts of the body and muscle belly, which suggests that the subjective pain in patients with knee OA is in the muscle. Studies have often suggested that the symptoms of knee OA are rather weakly associated with radiographic findings and vice versa [55]. However, this clinical trial demonstrated that functional changes in the MTrPs in the muscles are the main cause of knee pain. The main physiological effect of FSN on pain control is immediate and substantial pain relief at the point of muscle irritation, that is, improvement of the muscle where the MTrP is present. Because the abnormal contraction of the muscle segment worsens blood circulation, causing a lack of local oxygen and nutrients and the appearance of tight bands, blood circulation must be improved to relieve pain [56]. Muscles can compress blood vessels, obstructing blood circulation and nerves, affecting nerve conduction and bones, and causing bony changes that can limit movement and produce pain and paralysis. By eliminating the MTrP in the muscle, the pressure and constriction of blood vessels can be relieved, and local blood circulation and metabolism can be increased, enabling damaged local bodies to recover. In addition to the elimination of muscle pain, reduction in muscle contraction, and improvement of local circulation, reperfusion is a key element of FSN therapy for pain. Repeated stretching and contraction of the associated muscles through the reperfusion approach improve local circulation in damaged musculoskeletal tissues and modify ischemia and hypoxia at the point of myalgia [57]. However, during treatment, if the MTrP in the muscle is relieved but local tissues have not fully recovered, the state before treatment may recur. After resting and returning to normal metabolism, MTrPs can be completely eliminated. However, if other recurring factors appear during the process, the MTrP may return (Figure 9).

### 4.4. Limitations of This Study

This study had several limitations. First, because of differences in gender, age, and the location of bone growth, subgroup analysis may be required to determine the differences in the effectiveness of FSN therapy. Second, because the VAS, WOMAC, and Lequesne indices are subjective, the patients’ educational background and cognitive ability may have affected the results and produced bias in the outcome indicators. Third, the placebo and nocebo effects from the interventions in the clinical trial could have had repercussions for the patients and given rise to an improvement in symptoms. The insufficient placebo intervention should be modified in future clinically relevant trials of FSN, including both the swaying movement (passive treatment) and the reperfusion approach (active treatment). TENS is a totally passive treatment and lacks active treatment, so we will try to contract the leg muscles to make the procedures as similar as possible to each other in future research. Finally, the inability to blind therapists and participants to the therapeutic schemes may have affected the final results. Future research is required to establish a better experimental design.

## 5. Conclusions

Degenerative knee OA is usually diagnosed via an imaging study in modern medicine but it is sometimes asymptomatic. Soft tissues surrounding the knee joint, including muscles, ligaments, and tendons, play a bigger role in symptomatic knee OA. The MTrP in the muscle related to the knee joint may be suspected as a factor of symptomatic knee OA, and alleviating knee pain, strengthening walking ability, and improving overall functional performance by eliminating the MTrP in the muscle after the FSN treatment were found to be effective in our study. The swaying movement technique and reperfusion approach are used to increase local blood circulation and decrease muscle tightness and spasms. Our results demonstrated that pain relief was the primary benefit of FSN and that the most significant positive correlation was between pain suppression and the increase in joint mobility. Although this study demonstrated the effectiveness of FSN in the treatment of soft-tissue pain in degenerative knee OA, the mechanism of relieving muscle-excited pain points through FSN and reperfusion remains unknown. Further research is required to elicit and analyze this mechanism by using experimental animal models and exploring key elements of myofascial pain treatment through a scientific approach from the perspective of empirical medicine, thereby providing a reliable basis to increase the clinical applicability of FSN therapy.

## Figures and Tables

**Table 1:** Baseline characteristics and clinical evaluation indicators of participants.

Characteristics	FSN (N = 15)	TENS (N = 16)	*p*-Value
Age (years old)	65.73 ± 6.79 (54–77)	62.81 ± 5.72 (54–72)	0.24
Sex (M/F)	4/11	6/10	0.70
Affected side (Left/Right)	9/6	8/8	0.58
Height (cm)	161.00 ± 6.11	158.69 ± 6.25	0.34
Weight (kg)	69.00 ± 13.17	59.50 ± 9.57	0.14
VAS (0–10)	5.80 ± 1.42	5.81 ± 0.91	0.98
WOMAC-Total (0–96)	27.80 ± 12.12	26.44 ± 12.54	0.61
Lequesne index (0–24)	10.13 ± 3.27	8.75 ± 2.35	0.26
PPT (N/cm^2^)			
Quadriceps muscle	74.73 ± 28.63	86.56 ± 24.35	0.41
Pes anserinus	66.53 ± 34.06	63.38 ± 17.76	0.84
Gastrocnemius muscle	88.47 ± 34.05	82.13 ± 24.97	0.89
TH (N)			
Quadriceps muscle	47.79 ± 10.04	45.39 ± 9.51	0.83
Pes anserinus	41.44 ± 9.94	37.80 ± 4.40	0.14
Gastrocnemius muscle	36.79 ± 6.30	35.15 ± 6.97	0.68
ROM (degrees)			
Active ROM	103.87 ± 11.17	112.63 ± 16.31	0.07
Passive ROM	125.80 ± 15.33	135.69 ± 16.80	0.06
Walking speed (cm/s)	32.80 ± 6.60	35.96 ± 3.20	0.11

Data are expressed as the mean ± SD; *p*-values were analyzed with an independent *t*-test. Abbreviations: FSN, Fu’s subcutaneous needling; TENS, transcutaneous electrical nerve stimulation; VAS, visual analog scale; WOMAC, Western Ontario and McMaster Universities Osteoarthritis Index; PPT, pain pressure threshold; TH, tissue hardness of muscle; ROM, range of motion.

**Table 4:** WOMAC and Lequesne indices compared between the FSN and TENS groups.

	Day 1	Day 8		Day 15	
	Pre-tx	1-Week Follow-Up	*p*-Value	2-Week Follow-Up	*p*-Value
WOMAC					
FSN group	27.80 ± 12.12	21.87 ± 10.50	<0.05 *	22.00 ± 11.06	<0.05 *
TENS group	26.44 ± 12.54	18.31 ± 15.02	<0.05 *	19.13 ± 16.64	<0.05 *
Lequesne index					
FSN group	10.13 ± 3.27	8.20 ± 3.12	<0.05 *	7.27 ± 2.95	<0.05 *
TENS group	8.75 ± 2.25	7.50 ± 2.57	<0.05 *	8.25 ± 3.05	0.44

Data are expressed as the mean ± SD; * indicates a significant difference, analyzed by a paired *t*-test. Abbreviations: FNS, Fu’s subcutaneous needling; TENS, transcutaneous electrical nerve stimulation; WOMAC, Western Ontario and McMaster Universities.

## Data Availability

The data that support the findings of this study are available from the corresponding author, upon reasonable request.
